# Serum Osteopontin Level Correlates with Carotid-Femoral Pulse Wave Velocity in Geriatric Persons

**DOI:** 10.1155/2014/570698

**Published:** 2014-07-15

**Authors:** Chung-Jen Lee, Ji-Hung Wang, Yu-Chih Chen, Mei-Ling Chen, Chiu-Fen Yang, Bang-Gee Hsu

**Affiliations:** ^1^Department of Nursing, Tzu Chi College of Technology, Hualien, Taiwan; ^2^Division of Cardiology, Buddhist Tzu Chi General Hospital, Hualien, Taiwan; ^3^School of Medicine, Tzu Chi University, Hualien, Taiwan; ^4^Division of Nephrology, Buddhist Tzu Chi General Hospital, No. 707, Section 3, Chung-Yang Road, Hualien 970, Taiwan

## Abstract

Osteopontin (OPN) is involved in the regulation of vascular calcification processes. The aim of this study was to evaluate the relationship between fasting serum OPN concentration and carotid-femoral pulse wave velocity (cfPWV) in geriatric persons. Fasting blood samples were obtained from 93 geriatric persons. cfPWV were performed by SphygmoCor system. Serum OPN levels were measured using a commercially available enzyme-linked immunosorbent assay. Geriatric adults who had diabetes (*P* = 0.007) or dyslipidemia (*P* = 0.029) had higher cfPWV levels than those without diabetes or dyslipidemia. The univariable linear regression analysis showed that age (*P* = 0.002), waist circumference (*P* = 0.048), body mass index (*P* = 0.004), systolic blood pressure (*P* = 0.001), diastolic blood pressure (*P* = 0.036), pulse pressure (*P* = 0.017), creatinine (*P* = 0.002), and log-OPN level (*P* = 0.001) were positively correlated with cfPWV levels, while the high-density lipoprotein cholesterol (HDL-cholesterol) level (*P* = 0.007) and glomerular filtration rate (*P* = 0.001) were negatively correlated with cfPWV levels among the geriatric adults. Multivariable forward stepwise linear regression analysis of the significant variables also showed that log-OPN (*β* = 0.233, *R* ^2^ = 0.123, regression coefficient: 1.868, *P* = 0.011) was still an independent predictor of cfPWV levels in geriatric persons.

## 1. Introduction

Arterial stiffness is increased when elastic properties of the arterial wall are reduced [1]. Recently, arterial stiffness has been recognized as an independent risk factor for cardiovascular morbidity and mortality [2]. Carotid-femoral pulse wave velocity (cfPWV), a measure of the intrinsic stiffness of the aortic wall, is a direct measurement of aortic stiffness and has been recommended as the gold-standard measurement for arterial stiffness [3] and is an independent predictor of cardiovascular mortality and morbidity in elderly subjects [4, 5].

Advancing age is associated with changes in structure and function of different segments of the vascular system and is the dominant risk factor for cardiovascular diseases [6, 7]. Age-related vascular changes include intimal and medial thickening, arterial calcification, and increased deposition of matrix substances, thus leading to a reduced compliance and increased arterial wall stiffness [6]. Arterial calcification in blood vessels is an active, cell-regulated process, in which mineralization is the net result of a transdifferentiation process of vascular smooth muscle cells to chondrocyte-like or osteoblast-like cells, and may lead to increased arterial stiffness [8]. Osteopontin (OPN) is a potent inhibitor of mineralization; it prevents ectopic calcium deposits and is a potent inducible inhibitor of vascular calcification [9]. In recent studies, OPN has been associated with increased cfPWV in rheumatoid arthritis patients [10], coronary artery disease patients [11], and healthy subjects [12]. It also has been hypothesized that OPN has a role in atherosclerosis, since elevated levels of OPN are associated with both the extent of cardiovascular disease, independent of traditional risk factors [13], and restenosis [14]. The aim of this study was to determine the relationship between fasting serum OPN levels and cfPWV among geriatric persons.

## 2. Materials and Methods

### 2.1. Participants

Between January and December 2012, 93 elderly volunteers aged 65 years or older at a medical center in Hualien, eastern Taiwan, were enrolled into this study. Trained staff measured blood pressure (BP) in the morning for all participants, using standard mercury sphygmomanometers with appropriate cuff sizes, after the participants had been sitting for at least 10 minutes. Systolic blood pressure (SBP) and diastolic blood pressure (DBP) were taken at the points of appearance and disappearance, respectively, of the Korotkoff sounds. SBP and DBP were taken three times at five-minute intervals and were averaged for analysis. In the prevalence survey, hypertension was defined as SBP ≥140 mmHg, and/or DBP ≥90 mmHg, or prescription of antihypertensive medication in the past two weeks. A person was regarded as diabetic if the fasting plasma glucose was either 126 mg/dL or more or if he/she was using diabetes medication (oral or insulin) [15]. Pulse pressure was calculated by subtracting DBP from SBP. Dyslipidemia was defined as triglycerides of 150 mg/dL or higher, high-density lipoprotein cholesterol (HDL-cholesterol) level less than 40 mg/dL in men or less than 50 mg/dL in women [16]. The Protection of the Human Subjects Institutional Review Board of Tzu-Chi University and Hospital approved this study. Participants were excluded if they had an acute infection, acute myocardial infarction, and pulmonary edema at the time of blood sampling, if they used calcium, active vitamin D metabolites, bisphosphonates, teriparatide, or estrogens medication, or if they declined to provide informed consent for the study.

### 2.2. Anthropometric Analysis

Patient weight was measured in light clothing and without shoes to the nearest 0.5 kilograms, and height was measured to the nearest 0.5 cm. Waist circumference was measured using a tape measurement around the waist from the point between the lowest ribs and the hip bones with the hands on the hips. Body mass index (BMI) was calculated as the weight in kilograms divided by the height in meters squared [17–19].

### 2.3. Biochemical Investigations

Fasting blood samples (approximately 5 mL) were immediately centrifuged at 3000 g for 10 min. Serum levels of blood urea nitrogen (BUN), creatinine (Cre), fasting glucose, total cholesterol (TCH), triglycerides (TG), HDL-cholesterol, low-density lipoprotein cholesterol (LDL-cholesterol), total calcium, phosphorus, and C-reactive protein (CRP) were measured using an autoanalyzer (COBAS Integra 800, Roche Diagnostics, Basel, Switzerland) [17–19]. Serum OPN levels (eBioscience Inc., San Diego, CA, USA) were measured using a commercial available enzyme-linked immunosorbent assay (ELISA). Serum intact parathyroid hormone levels (iPTH) (Diagnostic Systems Laboratories, Webster, Texas, USA) were measured using a commercially available ELISA [18]. The estimate glomerular filtration rate (GFR) calculation in this study used Modification of Diet in Renal Disease (MDRD) equation.

### 2.4. Carotid-Femoral Pulse Wave Velocity Measurements

Measurements of cfPWV were performed using a pressure tonometer to transcutaneously record the pressure pulse waveform in the underlying artery (SphygmoCor system, AtCor Medical, Australia), as previously described [17]. All measurements were performed in the morning in the supine position after a minimum 10 min rest in a quiet, temperature-controlled room. Records were made simultaneously with an ECG signal, which provided an *R*-timing reference. Pulse wave recordings were performed consecutively at two superficial artery sites (carotid-femoral segment). Integral software was used to process each set of pulse wave and ECG data to calculate the mean time difference between *R*-wave and pulse wave on a beat-to-beat basis, with an average of 10 consecutive cardiac cycles. The cfPWV was calculated using the distance and mean time difference between the two recorded points. Quality indices, included in the software, were set to ensure uniformity of data.

### 2.5. Statistical Analysis

Data are expressed as the mean ± standard deviation (SD) and were tested for normal distribution using Kolmogorov-Smirnov statistics. Comparisons between patients were performed using Student's independent *t*-test (two-tailed) for normally distributed data or the Mann-Whitney *U* test for parameters that presented a nonnormal distribution. Because the glucose, CRP, iPTH, and OPN were nonnormal distribution, we used a logarithm to base 10 and noted log-glucose, log-CRP, log-iPTH, and log-OPN were normal distribution. Clinical variables that correlated with cfPWV levels in hypertensive patients were evaluated by univariable linear regression analysis. Variables significantly associated with cfPWV in geriatric adults were tested for independency in multivariable forward stepwise regression analysis. Data were analyzed using SPSS for Windows (version 19.0; SPSS Inc., Chicago, IL, USA). A *P* value <0.05 was considered statistically significant.

## 3. Results

The clinical and laboratory characteristics of geriatric adults are presented in Tables 1 and 2. The medical histories of the geriatric adults included diabetes (*n* = 44 (47.3%)), dyslipidemia (*n* = 44 (47.3%)), and hypertension (*n* = 37 (39.8%)). The medications prescribed to the geriatric adults included angiotensin-receptor blocker (ARB; *n* = 26 (28.0%)), angiotensin-converting enzyme inhibitor (ACEI; *n* = 16 (17.2%)), calcium-channel blocker (CCB; *n* = 22 (23.7%)), *β*-blocker (*n* = 22 (23.7%)), statins (*n* = 23 (24.7%)), and fibrate (*n* = 18 (19.4%)). Geriatric adults who had diabetes had higher cfPWV levels than those without diabetes (*P* = 0.007). It is also noted that geriatric adults who had dyslipidemia had higher cfPWV levels than those without dyslipidemia (*P* = 0.029). The cfPWV levels did not differ statistically based on gender, coexisting hypertension, and ARB, ACEI, CCB, *β*-blocker, statins, and fibrate use.

The univariable linear analysis of cfPWV levels in geriatric adults is presented in Table 3. Age (*r* = 0.310; *P* = 0.02), waist circumference (*r* = 0.206; *P* = 0.048), BMI (*r* = 0.294; *P* = 0.004), SBP (*r* = 0.340; *P* = 0.001), DBP (*r* = 0.218; *P* = 0.036), pulse pressure (*r* = 0.247; *P* = 0.017), Cre (*r* = 0.317; *P* = 0.002), and log-OPN level (*r* = 0.351; *P* = 0.001) were positively correlated with cfPWV levels, while the HDL-cholesterol level (*r* = −0.279; *P* = 0.007) and GFR (*r* = −0.330; *P* = 0.001) were negatively correlated with cfPWV levels among the geriatric adults.

Multivariable forward stepwise linear regression analysis of the variables that were significantly associated with cfPWV levels (diabetes, dyslipidemia, age, waist circumference, BMI, SBP, DBP, pulse pressure, Cre, HDL-cholesterol, GFR, and log-OPN) among geriatric adults showed that log-OPN (*β* = 0.233, *R* ^2^ = 0.123, regression coefficient: 1.868, *P* = 0.011), BMI (*β* = 0.333, *R* ^2^ = 0.093, regression coefficient: 0.243, *P* < 0.001), Cre (*β* = 0.195, *R* ^2^ = 0.074, regression coefficient: 1.517, *P* = 0.033), age (*β* = 0.250, *R* ^2^ = 0.047, regression coefficient: 0.130, *P* = 0.007), and diabetes (*β* = 0.195, *R* ^2^ = 0.035, regression coefficient: 1.090, *P* = 0.030) were the independent predictors of cfPWV levels in geriatric adults (Table 4).

## 4. Discussion

The results of our study showed that diabetes, dyslipidemia, age, waist circumference, BMI, SBP, DBP, pulse pressure, Cre, and log-OPN level were positively correlated with cfPWV levels, while HDL-cholesterol level and GFR were negatively correlated with cfPWV levels among the geriatric adults. After adjusting significant variables by multivariable forward stepwise linear regression analysis, it was shown that log-OPN, BMI, Cre, age, and diabetes were the independent predictors of cfPWV levels in geriatric adults.

The aorta stiffens with aging, and decreased arterial compliance is one of the earliest detectable manifestations of adverse structural and functional changes within the vessel wall [20]. Pulse wave velocity is calculated as the distance traveled by a pulse wave divided by the time taken to travel the distance [21]. cfPWV is a direct measurement of aortic stiffness and has been recommended as the gold-standard measurement for arterial stiffness [3]. Diabetes is a disease of accelerated arterial ageing, as shown by stiffer arteries and consequently steeper increases in pulse pressure with age in these subjects [22]. A systematic review study noted diabetes mellitus was positively associated with cfPWV in 52% studies, but the strength of the association between cfPWV and diabetes mellitus was weak, with the presence of diabetes mellitus accounting for a mean of 5% of the variation in cfPWV [23]. Our study showed that diabetes mellitus was positively correlated with cfPWV among the geriatric adults. This association remained significant even after adjustment for various confounders in geriatric subjects and accounting for a 3.5% of the variation in cfPWV.

Aging of the arterial system is accompanied by progressive structural changes, consisting of fragmentation and degeneration of elastin, increases in collagen, thickening of the arterial wall, endothelium damage, and progressive dilation of the arteries [24]. Arterial stiffness leads to an increase in SBP because hearts are pumping into a stiffer arterial bed that is less able to accommodate the volume of blood pumped by the left ventricle, which results in a greater pressure increase in systole exposing the myocardium to higher SBP [22]. Reduced aortic elastic recoil and reservoir capacity lead to fall in DBP resulting in the widened pulse pressure [21]. A systematic review study noted age and blood pressure were well-established association of cfPWV (91% and 90% of studies, resp.) [23]. Our study showed that age, SBP, DBP, and pulse pressure were positively correlated with cfPWV among the geriatric subjects. In our study after the multivariable analysis, age was also an independent predictor of cfPWV in geriatric subjects.

Increased arterial stiffness associated with metabolic syndrome may in part explain the increased cardiovascular disease risk observed in these conditions [22]. Waist circumference was positively correlated with cfPWV, while BMI was not associated with cfPWV in a study of Chinese community-dwelling adults [25]. Increases in waist circumference were also independently associated with higher cfPWV among youth with Type 1 diabetes [26]. However, another study noted cfPWV correlated positively with BMI, but not waist circumference in the Brazilian population [27]. The conflicting data concerning the effects of waist circumference or BMI on outcomes of cfPWV may be caused by differences in study population, study design, age, and inclusion criteria. Patients with lecithin-cholesterol acyltransferase mutation noted had low HDL-cholesterol levels that increased arterial stiffness [28]. Our study showed that dyslipidemia, waist circumference, and BMI were positively correlated with cfPWV levels, while the HDL-cholesterol level was negatively correlated with cfPWV levels among the geriatric adults. BMI was also the independent predictor of cfPWV levels in geriatric subjects in our study after multivariable analysis.

There is evidence that cfPWV increases as GFR falls [29]. Decreased GFR exhibited a significant reverse association with cfPWV in women with normal to mildly impaired renal function [30]. Coronary artery disease patients with impaired renal function had greater cfPWV compared to those with coronary artery disease patients and normal renal function [31]. Our study showed that, among geriatric subjects, Cre was positively correlated with cfPWV levels, while the GFR was negatively correlated with cfPWV levels among the geriatric adults. In our study after multivariable analysis, Cre were also independent predictors of arterial stiffness among geriatric adults accounting for a 7.4% of the variation in cfPWV.

Arterial stiffness is associated with vascular calcification [8]. Vascular calcification is a consequence of tightly regulated processes that culminate in an organized extracellular matrix deposition by osteoblast-like cells [32]. OPN is a phosphorylated acidic glycoprotein adhesion molecule and plays a significant role in a variety of biological processes, including bone resorption, immune cell activation, inhibition of vascular calcification, and extracellular matrix remodeling [33]. OPN is a potent inhibitor of mineralization; it prevents ectopic calcium deposits and is a potent inducible inhibitor of vascular calcification [9]. Clinical studies suggest that serum OPN levels were positively associated with cfPWV in rheumatoid arthritis patients, coronary artery disease patients, and healthy subjects [10–12]. Our study showed that serum log-OPN concentrations were positively correlated with cfPWV levels among the geriatric adults. Multivariable forward stepwise linear regression analysis of the significant variables also showed that serum log-OPN concentration was also an independent predictor of cfPWV levels in the current study.

Pharmacologic interventions may influence cfPWV in humans [6]. There is some evidence that angiotensin-receptor blocker, angiotensin-converting enzyme inhibitors, calcium-channel blockers, beta-blocker, or diuretic reduced significantly cfPWV compared to a placebo beyond blood pressure-lowering effects [6, 34]. However, other studies showed that different antihypertension regimens produced clear differential effects on central aortic systolic and pulse pressures but little difference in pulse wave velocity between the treatments compared in the studies [35]. A systematic review paper also cannot safely conclude the effect of statins on arterial stiffness, as estimated by pulse wave velocity measurements [36]. Our results did not show a relationship between statins or fibrate or other medications (ARB, ACEI, CCB, *β*-blocker) and cfPWV levels among geriatric adults. Further studies are therefore required to elucidate the relationship between medication and cfPWV levels in geriatric subjects.

Our previous study noted serum osteoprotegerin (OPG) levels positively associated with cfPWV levels in hypertensive patients [17]. OPG is an essential secreted protein in bone turnover due to its role as a decoy receptor for the receptor activator of NF-*κ*B ligand (RANKL) in the osteoclasts, thus inhibiting their differentiation [37]. The vascular role of OPG is multifaceted and depends on the interplay with its ligands, RANKL and tumor necrosis factor- (TNF-) related apoptosis-inducing ligand (TRAIL), and a bidirectional modulation involving osteogenic, inflammatory, and apoptotic responses [38]. OPN is also a potent inducible inhibitor of vascular calcification [9]. In this study, we also noted OPN level was still an independent predictor of cfPWV levels in geriatric persons. The relationship of vascular calcification inhibitor between OPG and OPN in cfPWV levels needs further studies.

Our study had some limitations. First, this study had a cross-sectional design. Therefore, our findings should be investigated in long-term prospective studies before a causal relationship between serum OPN levels and cfPWV in geriatric adults can be established. Second, the study was restricted to a limited number of geriatric adults, so that the possibility of indication bias cannot be excluded. Further studies are needed to show the effects of OPN on cfPWV in geriatric adults.

In summary, we showed an independent association of cfPWV with log-OPN, BMI, Cre, age, and diabetes mellitus of clinical importance in geriatric adults which requires further investigation.

## Figures and Tables

**Table 1:** Clinical and analytical characteristics of 93 geriatric adults.

Item	Parameter		Parameter	
Anthropometric findings	Height (cm)	159.51 ± 8.21	Waist circumference (cm)	91.70 ± 10.94
	Body weight (kg)	64.62 ± 10.47	Age (year)	72.16 ± 5.39
	Body mass index (kg/m^2^)	25.43 ± 3.85	SBP (mmHg)	131.74 ± 18.13
	DBP (mmHg)	70.85 ± 7.38	Pulse pressure (mmHg)	60.89 ± 16.66

Biochemical findings	Triglyceride (mg/dL)	134.58 ± 76.34	Total cholesterol (mg/dL)	173.41 ± 36.05
	Fasting glucose (mg/dL)	127.31 ± 49.86	HDL-cholesterol (mg/dL)	47.15 ± 12.59
	LDL-cholesterol (mg/dL)	102.90 ± 33.72	Creatinine (mg/dL)	1.19 ± 0.36
	BUN (mg/dL)	18.13 ± 6.85	GFR (mL/min)	63.88 ± 20.16
	Total calcium (mg/dL)	9.10 ± 0.38	Phosphorus (mg/dL)	3.45 ± 0.48
	Ca × P product (mg^2^/dL^2^)	31.37 ± 4.56	iPTH (pg/mL)	58.83 ± 34.10
	CRP (mg/dL)	0.30 ± 0.37	Osteopontin (ng/mL)	280.16 ± 230.86

Data are expressed as means ± standard deviations.

SBP, systolic blood pressure; DBP, diastolic blood pressure; HDL-cholesterol, high-density lipoprotein cholesterol; LDL-cholesterol, low-density lipoprotein cholesterol; BUN, blood urea nitrogen; GFR, glomerular filtration rate; Ca × P product, calcium-phosphorus product; iPTH, intact parathyroid hormone; CRP, C-reactive protein.

**Table 2:** Clinical characteristics and carotid-femoral pulse wave velocity levels of 93 geriatric adults.

Characteristic		Number (%)	cfPWV (m/s)	*P* value
Gender	Male	63 (67.7)	10.07 ± 2.60	0.869
	Female	30 (32.3)	10.17 ± 3.25	

Diabetes	No	49 (52.7)	9.37 ± 2.52	0.007^∗^
	Yes	44 (47.3)	10.92 ± 2.92	

Dyslipidemia	No	49 (52.7)	9.50 ± 2.38	0.029^∗^
	Yes	44 (47.3)	10.77 ± 3.11	

Hypertension	No	56 (60.2)	9.93 ± 2.80	0.473
	Yes	37 (39.8)	10.36 ± 2.85	

ACE inhibitor	No	77 (82.8)	10.11 ± 2.83	0.973
	Yes	16 (17.2)	10.08 ± 2.79	

ARB	No	67 (72.0)	9.95 ± 2.80	0.409
	Yes	26 (28.0)	10.49 ± 2.85	

*β*-Blocker	No	71 (76.3)	10.07 ± 2.09	0.841
	Yes	22 (23.7)	10.21 ± 2.61	

CCB	No	71 (76.3)	9.98 ± 2.74	0.457
	Yes	22 (23.7)	10.49 ± 3.05	

Statins	No	70 (75.3)	10.19 ± 2.91	0.583
	Yes	23 (24.7)	9.82 ± 2.52	

Fibrate	No	75 (80.6)	10.11 ± 2.84	0.937
	Yes	18 (19.4)	10.06 ± 2.74	

Data are expressed as means ± standard deviations.

^*∗*^ *P* < 0.05 was considered statistically significant after Student's *t*-test.

cfPWV, carotid-femoral pulse wave velocity; CAD, coronary artery disease; CHF, congestive heart failure; ACE, angiotensin-converting enzyme; ARB, angiotensin-receptor blocker; CCB, calcium-channel blocker.

**Table 3:** Correlation of carotid-femoral pulse wave velocity and clinical variables by univariable linear regression analysis among the 93 geriatric adults.

Variable	*R* value	*P* value
Age (years)	0.310	0.002^∗^
Height (cm)	−0.167	0.110
Body weight (kg)	0.157	0.133
Waist circumference (cm)	0.206	0.048^∗^
Body mass index (kg/m^2^)	0.294	0.004^∗^
Systolic blood pressure (mmHg)	0.340	0.001^∗^
Diastolic blood pressure (mmHg)	0.218	0.036^∗^
Pulse pressure (mmHg)	0.247	0.017^∗^
Total cholesterol (mg/dL)	−0.083	0.427
Triglyceride (mg/dL)	0.050	0.635
HDL-C (mg/dL)	−0.279	0.007^∗^
LDL-C (mg/dL)	0.064	0.545
Log-glucose (mg/dL)	0.066	0.531
BUN (mg/dL)	0.163	0.118
Creatinine (mg/dL)	0.317	0.002^∗^
GFR (mL/min)	−0.330	0.001^∗^
Log-CRP (mg/dL)	0.106	0.316
Total calcium (mg/dL)	0.030	0.776
Phosphorus (mg/dL)	−0.002	0.986
Ca × P product (mg^2^/dL^2^)	0.009	0.932
Log-iPTH (pg/mL)	0.204	0.050
Log-OPN (pg/mL)	0.351	0.001^∗^

^*∗*^ *P* < 0.05 is considered statistically significant in the univariable linear analyses.

Data of fasting glucose, CRP, iPTH, and OPN levels showed skewed distribution and therefore were log-transformed before analysis.

BUN, blood urea nitrogen; HDL-C, high-density lipoprotein cholesterol; LDL-C, low-density lipoprotein cholesterol; GFR, glomerular filtration rate; CRP, C-reactive protein; Ca × P product, calcium-phosphorus product; iPTH, intact parathyroid hormone; OPN, osteopontin.

**Table 4:** Multivariable stepwise linear regression analysis of carotid-femoral pulse wave velocity among 93 geriatric adults.

Items	Beta	*R* ^2^ change	Regression coefficient (95% confidence interval)	*P* value
Log-OPN (pg/mL)	0.233	0.123	1.868 (0.444 to 3.291)	0.011^∗^
Body mass index (kg/m^2^)	0.333	0.093	0.243 (0.119 to −0.367)	<0.001^∗^
Creatinine (mg/dL)	0.195	0.074	1.517 (0.123 to 2.911)	0.033^∗^
Age (years)	0.250	0.047	0.130 (0.037 to 0.223)	0.007^∗^
Diabetes	0.195	0.035	1.090 (0.108 to 2.072)	0.030^∗^

Data of osteopontin levels showed skewed distribution and therefore were log-transformed before analysis.

^*∗*^ *P* < 0.05 was considered statistically significant in the multivariable stepwise linear regression analysis (adopted factors: diabetes, dyslipidemia, age, waist circumference, body mass index, systolic blood pressure, diastolic blood pressure, pulse pressure, HDL-C, creatinine, GFR, and log-OPN).

HDL-C, high-density lipoprotein cholesterol; GFR, glomerular filtration rate; OPN, osteopontin.
